# Evaluation of procalcitonin-guided antimicrobial stewardship in patients admitted to hospital with COVID-19 pneumonia

**DOI:** 10.1093/jacamr/dlab133

**Published:** 2021-08-20

**Authors:** Maria Calderon, Ang Li, Juan Carlos Bazo-Alvarez, Jonathan Dennis, Kenneth F Baker, Ina Schim van der Loeff, Aidan T Hanrath, Richard Capstick, Brendan A I Payne, Daniel Weiand, Ewan Hunter, Ulrich Schwab

**Affiliations:** 1 Department of Infection and Tropical Medicine, The Newcastle upon Tyne Hospitals NHS Foundation Trust, Royal Victoria Infirmary, Newcastle upon Tyne, UK; 2 Research Department of Primary Care & Population Health, University College London, London, UK; 3 Escuela de Medicina, Universidad Cesar Vallejo, Trujillo, Peru; 4 Translational and Clinical Research Institute, Newcastle University, Newcastle upon Tyne, UK; 5 NIHR Newcastle Biomedical Research Centre, Newcastle University and The Newcastle upon Tyne Hospitals NHS Foundation Trust, Newcastle upon Tyne, UK; 6 Microbiology Department, Integrated Laboratory Medicine Directorate, The Newcastle upon Tyne Hospitals NHS Foundation Trust, Newcastle upon Tyne, UK

## Abstract

**Background:**

Procalcitonin is a biomarker that may be able to identify patients with COVID-19 pneumonia who do not require antimicrobials for bacterial respiratory tract co-infections.

**Objectives:**

To evaluate the safety and effectiveness of a procalcitonin-guided algorithm in rationalizing empirical antimicrobial prescriptions in non-critically ill patients with COVID-19 pneumonia.

**Methods:**

Retrospective, single-site, cohort study in adults hospitalized with confirmed or suspected COVID-19 pneumonia and receiving empirical antimicrobials for potential bacterial respiratory tract co-infection. Regression models were used to compare the following outcomes in patients with and without procalcitonin testing within 72 h of starting antimicrobials: antimicrobial consumption (DDD); antimicrobial duration; a composite safety outcome of death, admission to HDU/ICU or readmission to hospital within 30 days; and length of admission. Procalcitonin levels of ≤0.25 ng/L were interpreted as negatively predictive of bacterial co-infection. Effects were expressed as ratios of means (ROM) or prevalence ratios (PR) accordingly.

**Results:**

259 patients were included in the final analysis. Antimicrobial use was lower in patients who had procalcitonin measured within 72 h of starting antimicrobials: mean antimicrobial duration 4.4 versus 5.4 days, adjusted ROM 0.7 (95% CI 0.6–0.9); mean antimicrobial consumption 6.8 versus 8.4 DDD, adjusted ROM 0.7 (95% CI 0.6–0.8). Both groups had similar composite safety outcomes (adjusted PR 0.9; 95% CI 0.6–1.3) and lengths of admission (adjusted ROM 1.3; 95% CI 0.9–1.6).

**Conclusions:**

A procalcitonin-guided algorithm may allow for the safe reduction of antimicrobial usage in hospitalized non-critically ill patients with COVID-19 pneumonia.

## Introduction

It can be difficult to confidently distinguish COVID-19 pneumonia from bacterial pneumonia, owing to similarities in clinical presentation and frequently raised inflammatory markers including C-reactive protein.^1–3^ Furthermore, there are few data on how frequently bacterial co-infection occurs in patients with COVID-19. Although a large proportion of patients admitted to hospital with COVID-19 pneumonia receive antimicrobials [83% overall, 93% of patients admitted to a high dependency unit (HDU) or ICU],^2^^,^^4^ rates of confirmed bacterial co-infection are low (6.9% overall, 8.1% in critically unwell patients^5^^,^^6^), suggesting that antimicrobials are not necessary for most patients. Judicious use of antimicrobials is an important element of efforts to limit rising antimicrobial resistance worldwide,^7^ and the widespread use of antimicrobials for COVID-19 pneumonia is therefore of concern.^8^ Biomarkers or decision-aids that can assist clinicians in distinguishing COVID-19 pneumonia from bacterial community-acquired pneumonia (CAP) are therefore needed. Previous publications have postulated procalcitonin as a possible useful aid for antimicrobial stewardship in COVID-19 pneumonia, especially in light of the indiscriminate antimicrobial usage.^9–11^

Procalcitonin is a protein precursor of calcitonin that is present in low levels in healthy individuals.^12^ It is modulated by IL-1β, TNF-α, IL-2 and IL-6, and while levels rise in infection in general, the response to cytokines involved in viral infection is less marked.^13^^,^^14^ Procalcitonin-based protocols for limiting antimicrobial use for acute respiratory tract infections have been evaluated in a Cochrane systematic review.^15^ This showed statistically significant reductions in antimicrobial exposure and antimicrobial-associated side effects compared with standard care, with no differences in rates of treatment failure.

In the present study, we evaluated the effectiveness and safety of a procalcitonin-guided antimicrobial decision-aid in non-critically ill patients with COVID-19 pneumonia.

## Patients and methods

### Ethics

This project was conducted in accordance with the Declaration of Helsinki and national and institutional standards. As a retrospective service evaluation, formal ethical approval was not required. Patient consent was not required as a study of COVID-19 in accordance with Regulation 3(4) of the Health Service Control of Patient Information Regulations 2002.^16^ Approval for relevant patient-identifiable information to be used was granted by the Newcastle upon Tyne Hospitals (NUTH) Caldicott Guardian.

### Study design and setting

We conducted a retrospective, single-site cohort study to evaluate the clinical effectiveness and safety of a procalcitonin-guided protocol on the use of empirical antimicrobials in patients admitted to the NUTH NHS Foundation Trust, UK, with confirmed or suspected COVID-19 pneumonia. NUTH provides secondary and tertiary care across two hospital sites with over 1800 inpatient beds. It is also one of five centres in England designated for the management of airborne high consequence infectious diseases.^17^ Procalcitonin assays were not routinely performed at NUTH prior to the COVID-19 pandemic and were introduced as part of the emergency response.

We report our findings using the recommendations of the Strengthening the Reporting of Observational Studies in Epidemiology (STROBE) initiative^18^ (see Section 1 of the Supplementary data at *JAC* Online).

### Inclusion and exclusion criteria

Patients admitted to NUTH with laboratory-confirmed or clinically suspected COVID-19 pneumonia between 12/03/2020 and 01/07/2020 were identified through clinical coding and laboratory record searches. These dates encompass the period immediately following the formal end of the ‘contain’ phase of the UK pandemic response, capturing the first wave of the UK epidemic.^19^ Cases of COVID-19 pneumonia were defined as patients with clinical or radiological evidence of pneumonia and either a positive SARS-CoV-2 PCR swab taken during admission, or an admission episode with ICD-10 code U07.2.^20^ This code encompasses cases that have been diagnosed on clinical or epidemiological grounds but in which the virus has not been detected.

Electronic case notes including observations, investigation results and prescription records were reviewed. Patients were excluded for any of the following reasons: being younger than 18 years; being admitted to the HDU or ICU within 72 h of admission; not receiving antimicrobials during their admission; indication for antimicrobial treatment other than respiratory tract infection; antimicrobials being stopped as part of treatment rationalization in end-of-life care. A sensitivity analysis that included this final group of patients was performed to reflect real world practice, exploring whether exclusion of these patients led to a change in the overall findings.

Included patients were grouped for comparison according to whether or not procalcitonin was measured within 72 h after antimicrobial initiation. The upper limit of 72 h was chosen in line with Public Health England’s ‘Start Smart—Then Focus’ antimicrobial stewardship toolkit.^21^

The following data were collected for all included patients: demographics; comorbidities of cardiovascular disease, respiratory disease, diabetes and obesity; duration of symptoms prior to admission; symptoms of cough, shortness of breath, fever and gastrointestinal symptoms; vital sign observations; and radiological findings on admission. Prescribing data on antimicrobial indication, dose, route, and duration were collected; any antimicrobials continued after discharge were assumed to have been completed as prescribed. If patients participated in open label clinical trials and were randomized to receive antimicrobials as an investigational product, these treatments were not included in estimates of antimicrobial exposure or consumption. Any other antimicrobials received by these patients for respiratory tract infection were included in the analysis. Any available results from culture of respiratory and/or blood samples were also reviewed and collated.

### Procalcitonin assay

The Cobas Elecsys BRAHMS Procalcitonin immunoassay was introduced on 30 March 2020 for quantitative determination of procalcitonin;^22^ serum for this assay was collected in standard BD vacutainer^®^ SST II™ Advance tubes. Introduction of the assay was accompanied by dissemination of clinical guidance containing an institutional algorithm describing interpretation of procalcitonin results. This recommended that empirical antimicrobial treatment for respiratory tract infection could be stopped in patients with proven or suspected SARS-CoV-2 infection who had a procalcitonin level of ≤0.25 ng/mL. This cut-off was chosen to correspond with a conservative approach in a ‘moderate risk/acuity’ group of hospitalized patients.^23^ Results were not interpreted in isolation but rather as an adjunct to overall clinical judgement. A summary of the guidance is provided as Supplementary data (Section 2).

### Outcome measures

Primary outcome measures were duration of antimicrobial exposure and per-patient antimicrobial consumption. Duration of exposure was calculated as the time from first to last dose plus the prescribed dosing interval. Per-patient consumption was calculated by applying the current World Health Organisation Defined Daily Dose (DDD) method.^24^

To assess safety, we used a composite outcome comprising death, admission to HDU/ICU >72 h after starting antimicrobial treatment, or readmission to hospital within 30 days. Deaths in the community after discharge from hospital were also captured through robust daily electronic system record updates via primary care.

### Data analysis

Sample size was calculated based on the results of a systematic review by Schuetz *et al.*^15^ that evaluated procalcitonin-guided antimicrobial stewardship algorithms in patients with acute respiratory tract infections. To detect a similar reduction in antimicrobial exposure of 2.4 days (95% CI 2.15–2.71) with 80% power and 95% confidence would require at least 116 patients in each comparison group.^25^

Primary outcomes between the procalcitonin and non-procalcitonin groups were compared using ratios of means (ROM) and prevalence ratios (PR). ROMs were estimated for continuous outcomes by fitting generalized linear models (GLMs) with a logarithmic link and assuming a gamma distribution (γGLM). Dichotomous outcomes were compared using PR, which were estimated by fitting robust Poisson models. Adjusted estimates controlled for clinically plausible confounding variables identified in the univariate analysis and not found to possess collinearity with other confounders. Analyses with details of the models and confounders are detailed in Supplementary data (Section 3).

A time series analysis was performed to visualize antimicrobial consumption over time in both groups. Daily antimicrobial consumption by all patients included in the main analysis was aggregated; independent time series were then modelled for each group by fitting a GLM (Gaussian distribution, link identity), treating antimicrobial consumption as a continuous outcome and date of admission as a predictor. All models were adjusted for autocorrelation and seasonality using Fourier terms.

Reflecting the real-world effect of the availability and use of the procalcitonin assay, a sensitivity analysis was performed by re-including in both groups patients who had discontinued antimicrobials due to palliation at the end of life. Sensitivity analyses were performed for both the primary outcomes and time series analysis; a complete description is given in Supplementary data (Section 3). It is important to note that although some patients in the procalcitonin group were admitted just before the introduction of the procalcitonin assay, these individuals were able to have procalcitonin measured within the 72 h window, making them eligible for inclusion in the study.

All statistical analyses were performed using STATA 16.0 (StataCorp. 2011. Stata Statistical Software: Release 16. College Station, TX: StataCorp LP).

## Results

**Figure 1.**
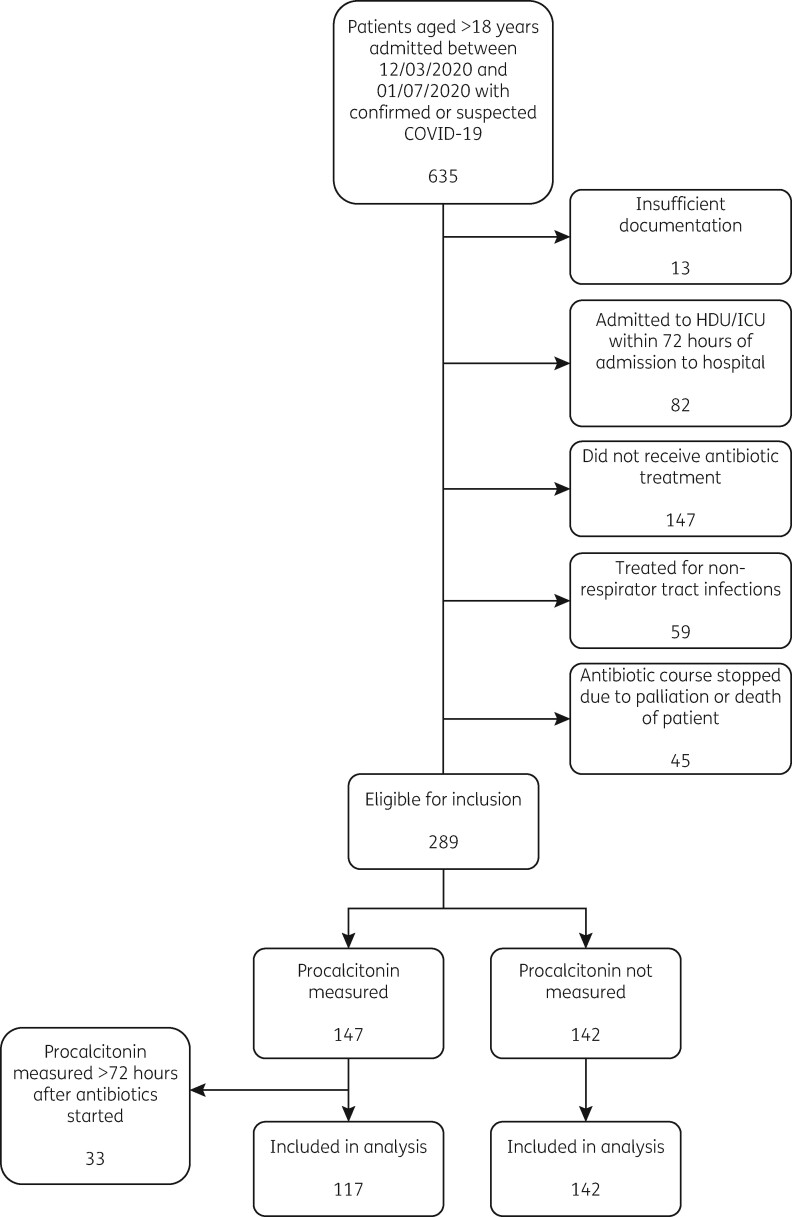
Flow chart of eligibility criteria.

635 patients were identified of whom 259 were retained for analysis after assessment against eligibility criteria. Of these, 117 (45.2%) had procalcitonin measured within 72 h after antimicrobial initiation (Figure 1).

The main characteristics of the study population are summarized in Table 1. After analysing the data, the following variables were included as potential confounders in the final model: age, comorbidities, COVID-19 status (laboratory confirmed versus clinically suspected), symptoms of fever or cough, need for supplemental oxygen on admission, tachypnoea at admission. Given the recent report by Williamson *et al.*^26^ that male sex was strongly associated with COVID-19-related death, sex was also included in the final model.

**Table 1.** Main characteristics of the population

Characteristic	Non-procalcitonin (*N = *142)	Procalcitonin (*N = *117)
Age, years, mean (SD)^a^	73.6 (±14.5)	67.7 (±17.0)
Age group, years, *n* (%)^a^		
18–39	5 (3.5)	8 (6.8)
40–49	6 (4.2)	11 (9.4)
50–59	14 (9.9)	18 (15.4)
60–69	15 (10.6)	27 (23.1)
70–79	46 (32.4)	18 (15.4)
>80	56 (39.4)	35 (29.9)
Sex, *n* (%)		
Female	70 (49.3)	56 (47.8)
Comorbidities, *n* (%)		
Any cardiovascular disease	83 (58.5)	59 (50.4)
Respiratory condition	46 (33.4)	46 (39.3)
Obesity	15 (10.6)	21 (17.9)
Diabetes (Type 1 or 2)	24 (16.9)	30 (25.6)
Number of comorbidities, *n* (%)^a^		
No comorbidities	12 (8.5)	11 (9.4)
1 to 2 comorbidities	38 (26.7)	27 (23.1)
More than 2 comorbidities	92 (64.8)	79 (67.5)
Clinical markers of severity		
Symptoms at admission, *n* (%)		
Fever^a^	58 (40.9)	72 (61.5)
Cough^a^	68 (47.9)	77 (65.8)
Shortness of breath	81 (57.0)	80 (68.4)
Gastrointestinal symptoms	11 (7.6)	18 (15.4)
Signs at admission, *n* (%)		
Fever (≥37.5°C)	49 (34.5)	48 (41.0)
Tachypnoea (>20 breaths/min)^a^	72 (50.7)	79 (67.5)
Oxygen supplementation^a^	59 (41.5)	67 (57.3)
NIV (on admission)	0 (0)	0 (0)
Time from onset of symptoms to admission, days, median (IQR)	5 (2–10)	3 (2–9)
Radiological changes at admission, *n* (%)	93 (65.5)	87 (74.4)
PCR-confirmed COVID-19, *n* (%)^a^	120 (84.5)	79 (67.5)
Procalcitonin levels, ng/mL, median (IQR)	–	0.2 (0.1–0.4)
Procalcitonin group, ng/mL, *n* (%)		
≤0.25	–	73 (62.4)
0.26–0.5	–	17 (14.5)
0.6–1.0	–	14 (12.0)
>1.0	–	13 (11.1)

NIV, non-invasive ventilation.

a Variables with statistical evidence of association that were included in the final model (*P < *0.05). Definitions of the variables included are described in Supplementary Material 3.

Patients in the procalcitonin group had lower antimicrobial exposure and consumption compared with those in the non-procalcitonin group (Table 2). In the procalcitonin group, the adjusted mean duration of antimicrobial therapy was 30% lower (adjusted ROM = 0.70; 95% CI: 0.6–0.9), as was the adjusted mean DDD (ROM = 0.70; 95% CI: 0.6–0.8; Table 2). DDDs for each antimicrobial, including route and combination, are summarized in Table S1. Microbiology results for each group are summarized in Table S2, with low rates of positive blood or sputum cultures in both groups.

**Table 2.** Summary of outcome measures and estimates of effect

Characteristic	Non-procalcitonin (*N = *142)	Procalcitonin (*N = *117)	Estimates of effect^a^	
			Unadjusted	Adjusted^b^
*Continuous measures*	Mean (SD)	Mean (SD)	ROM (95% CI)	ROM (95% CI)
Duration of antibiotics (days)	5.4 (2.8)	4.4 (3.1)	0.8 (0.7–0.9)	0.7 (0.6–0.9)
Consumption of antibiotic (DDD)	8.4 (5.7)	6.8 (5.6)	0.8 (0.7–0.9)	0.7 (0.6–0.8)
Length of stay (days)	9.1 (8.5)	9.8 (7.4)	1.1 (0.9–1.3)	1.3 (0.9–1.6)
*Categorical measures*	*n* (%)	*n* (%)	PR (95% CI)	PR (95% CI)
Composite safety outcome^c^	40 (28.1)	26 (22.2)	0.8 (0.6–1.2)	0.9 (0.6–1.3)
30 day mortality	27 (19.0)	8 (6.9)	0.5 (0.3–0.9)	0.6 (0.4–1.1)

ROM, ratio of means; PR, prevalence ratio; DDD, defined daily doses.

a Full details of modelling and variables are included in Supplementary Materials.

b Adjusted for age, sex, comorbidities, COVID-19 status, and fever, cough, respiratory rate and oxygen requirement at time of admission.

c This measure comprises death during admission, not being alive at 30 days from admission, readmission during study period, and/or admission to ICU.

There were no differences in the composite safety outcome in both unadjusted and adjusted analyses. Analysis of mortality in isolation showed a non-statistically significant trend towards patients in the procalcitonin group being less likely to die within 30 days (adjusted PR = 0.6; 95% CI: 0.4–1.1; Table 2).

**Figure 2.**
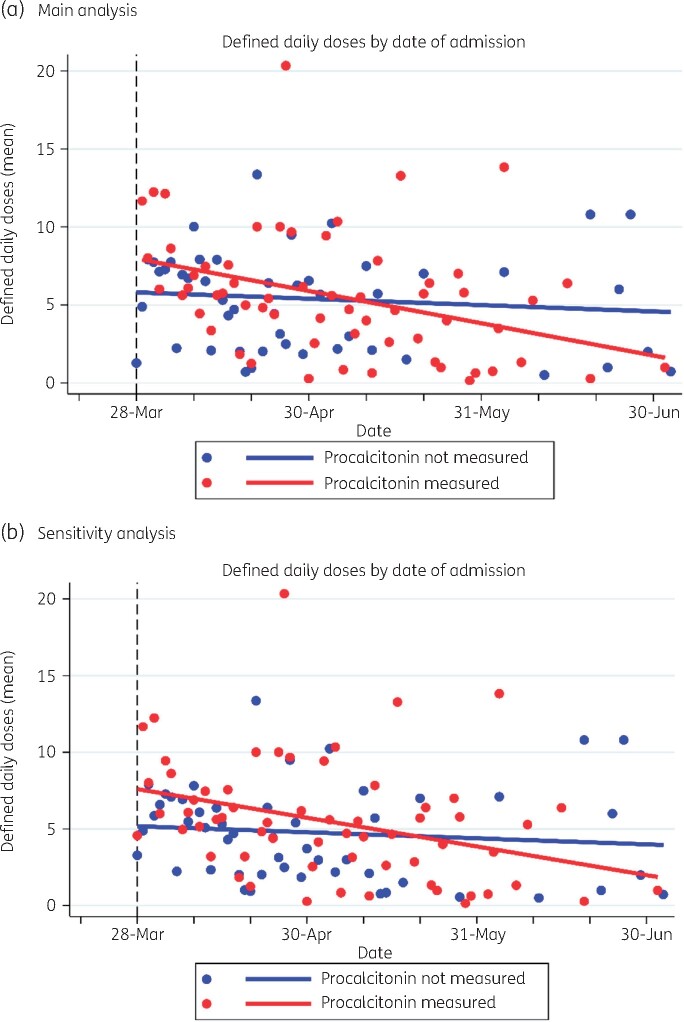
Time series analysis of antimicrobial consumption (DDD) over time. (a) Main analysis. (b) Sensitivity analysis including end-of life patients. Observation was considered from 28 March 2020, this was the earliest date of admission where patients fulfilled the inclusion criteria of procalcitonin measurement within 72 h of starting antimicrobials.

Time series analysis of the mean patient antimicrobial consumption showed a decreasing trajectory over time in the procalcitonin group (βslope=−0.07, 95% CI: −0.11 to −0.03), with negligible change seen in the non-procalcitonin group (βslope=−0.01, 95% CI: −0.05 to 0.02) (Figure 2a). Sensitivity analysis, after inclusion of those patients where antimicrobials were discontinued as part of end-of-life care, demonstrated similar findings (procalcitonin group: βslope=−0.06, 95% CI: −0.10 to −0.02; non-procalcitonin group: βslope=−0.01, 95% CI: −0.05 to 0.02) (Figure 2b). Although mean consumption was initially higher in the procalcitonin group, there was crossover in both primary and sensitivity analyses at between 1 and 2 months after assay introduction.

## Discussion

To our knowledge, this is the first study to evaluate the clinical impact and safety of early procalcitonin-guided antimicrobial stewardship in patients with COVID-19 pneumonia in comparison with a standard of care not including routine procalcitonin assays. Prior studies have been conducted within cohorts where all patients underwent procalcitonin testing,^27^ or have focused on the role of procalcitonin as a biomarker of disease severity and prognosis.^28^ We found a moderate reduction in antimicrobial duration and consumption in patients where procalcitonin was measured within 72 h of starting antimicrobials, with no differences in adverse outcomes or length of stay. Our results suggest that it is safe to use a procalcitonin-based protocol in the antimicrobial decision-making process. It is important to note that our algorithm used a cut-off of ≤0.25 ng/L as being negatively predictive of bacterial co-infection, and the potential impact of using higher or lower thresholds warrants evaluation in larger, ideally prospective, studies. Microbiological results from sputum and blood cultures are in keeping with the low frequency of isolates seen in comparable settings.^29^^,^^30^ Of note, all blood culture isolates from patients in either group were organisms that were regarded as contaminants.

A major advantage of the procalcitonin assay is that it can be rapidly performed using a readily obtainable blood sample, in contrast to respiratory tract cultures which are limited by challenges in sample collection and minimum growth times.^31^ There are, however, clinical situations that increase the potential for false-negative procalcitonin results, including localized infections such as empyema, atypical infections, patients receiving renal replacement therapy, and if measured too early or too late in the course of infection. These and other factors mandate the interpretation of procalcitonin levels within the context of other laboratory and clinical findings.^32^

The time series analysis demonstrates that antimicrobial consumption within the procalcitonin group reduced over time, diverging from consumption in the non-procalcitonin group at just over a month following introduction of the assay. We hypothesize that this may be explained by a relative lack of prior institutional experience with procalcitonin, with an initial period of adoption into practice at least partly accounting for this trend.

Our study has some important strengths. The number of patients within the comparison groups exceeded our *pre hoc* sample size calculation for comparison of antimicrobial durations. We also suggest that our safety outcome was robust, as we included all deaths within 30 days of starting antimicrobials, including community deaths following discharge from hospital.

There are also limitations that warrant discussion. While the WHO DDD method provides a standardized framework to measure antimicrobial consumption, there are limitations to this method in respect to antimicrobial stewardship goals. These include assigning equivalent importance to all classes, with no weighting applied to broad- versus narrow-spectrum agents. Furthermore, DDD does not account for dose adjustments in patients with low renal function or low body weight. Although an interrupted time series analysis was possible, the brevity of the period prior to introduction of the procalcitonin assay precludes drawing any firm inferences due to limited numbers of patients admitted with COVID-19 pneumonia. It is possible, however, to see trends over time that give an informative picture of changes that occurred after the assay was introduced. Another limitation of time series analyses is their inability to adjust for potential confounders, although the overall results are concordant with the γGLM-adjusted effect estimate for antimicrobial consumption. A description of socio-economic risk factors for adverse COVID-19 outcomes, as identified by Public Health England, would have added interest to our discussion. Our system does not routinely collect information on factors such as multiple deprivation index and these were therefore not available for this analysis. We did consider constructing a proxy socio-economic variable derived from area of residence, although this method is better suited to much larger cohort or ecological studies that are able to provide greater statistical power, and we decided that this approach would not be valid in a relatively small retrospective clinical study such as ours.^33^

Finally, the retrospective design may have introduced biases, thereby limiting inferences and extrapolation from our results. Selection bias is an important limitation inherent to observational studies like ours. For example, clinicians may have limited their use of the procalcitonin assay in patients in whom the presence of bacterial infection seemed less likely, such as those without clear clinical signs and symptoms suggestive of bacterial pneumonia. It is therefore possible that there are different *a priori* risks for the safety outcomes between the comparison groups. Similarly, the higher crude 30 day mortality rate within the non-procalcitonin group may be due to such differences, especially the unequal distribution of ages, a well-defined risk factor for mortality, between the groups.^34^ It was therefore unsurprising that there was no difference in 30 day mortality following adjustment for confounding.

We recognize that the care of patients with COVID-19 pneumonia has evolved over the course of the pandemic. For example, greater emphasis on non-invasive respiratory support with high flow nasal cannula or continuous positive airway pressure ventilation has meant that patients who previously required early transfer to HDU/ICU may now be cared for in specialized medical wards.^35^ Additionally, the majority of the patient cohort were admitted prior to the routine use of dexamethasone (22 June 2020) and remdesivir (5 June 2020) for patients with COVID-19 in the UK, potentially impacting the extrapolation of the mortality and safety outcomes that we found.^36^ Our study does not specifically identify patients who required early initiation of respiratory support, and the results should be interpreted with this in mind. We also excluded patients requiring early admission to HDU/ICU as we considered the clinical and microbiological risk factors in this group to be sufficiently different as to require independent study. Other groups have reported on the use of procalcitonin within this cohort of patients, demonstrating fewer days of antimicrobial use in patients with procalcitonin levels of <0.5 ng/mL.^27^

Finally, while our results support early procalcitonin testing as an antimicrobial stewardship tool, the economic implications of more widespread use also need to be considered. Given that the average cost of procalcitonin is £13.79 per test,^37^ evaluation of the cost-effectiveness of a procalcitonin-guided approach alongside other antimicrobial stewardship methods is required.

In conclusion, we observed that patients who underwent early procalcitonin measurement (within 72 h), supported by an algorithm to aid interpretation, had reduced empirical antimicrobial usage with no adverse effect on safety. Our finding supports the use and further investigation of procalcitonin as an antimicrobial stewardship tool for non-critically ill patients admitted with suspected or laboratory-confirmed COVID-19 pneumonia.

## Supplementary Material

dlab133_Supplementary_Data
