# Supplementary material for: Evaluation of procalcitonin-guided antimicrobial stewardship in patients admitted to hospital with COVID-19 pneumonia
Source: JAC Antimicrob Resist. 2021 Aug 20;3(3):dlab133. doi: 10.1093/jacamr/dlab133 (PMC8378277; doi:10.1093/jacamr/dlab133)
Supplement: dlab133_Supplementary_Data [file dlab133_supplementary_data.docx]

**Supplementary data**

**Section 1: STROBE Statement—checklist of items that should be included in reports of observational studies**

|  | | Item No | | Recommendation | | Check |
| --- | --- | --- | --- | --- | --- | --- |
| **Title and abstract** | | 1 | | (*a*) Indicate the study’s design with a commonly used term in the title or the abstract | | Yes |
|  |  |  |  | (*b*) Provide in the abstract an informative and balanced summary of what was done and what was found | | Yes |
| Introduction | | | | | |  |
| Background/rationale | | 2 | | Explain the scientific background and rationale for the investigation being reported | | Yes |
| Objectives | | 3 | | State specific objectives, including any prespecified hypotheses | | Yes |
| Methods | | | | | |  |
| Study design | | 4 | | Present key elements of study design early in the paper | | Yes |
| Setting | | 5 | | Describe the setting, locations, and relevant dates, including periods of recruitment, exposure, follow-up, and data collection | | Yes |
| Participants | | 6 | | (*a*) *Cohort study*—Give the eligibility criteria, and the sources and methods of selection of participants. Describe methods of follow-up  *Case-control study*—Give the eligibility criteria, and the sources and methods of case ascertainment and control selection. Give the rationale for the choice of cases and controls  *Cross-sectional study*—Give the eligibility criteria, and the sources and methods of selection of participants | | Yes |
|  |  |  |  | (*b*) *Cohort study*—For matched studies, give matching criteria and number of exposed and unexposed  *Case-control study*—For matched studies, give matching criteria and the number of controls per case | | NA |
| Variables | | 7 | | Clearly define all outcomes, exposures, predictors, potential confounders, and effect modifiers. Give diagnostic criteria, if applicable | | Yes |
| Data sources/ measurement | | 8* | | For each variable of interest, give sources of data and details of methods of assessment (measurement). Describe comparability of assessment methods if there is more than one group | | *Yes* |
| Bias | | 9 | | Describe any efforts to address potential sources of bias | |  |
| Study size | | 10 | | Explain how the study size was arrived at | | Yes |
| Quantitative variables | | 11 | | Explain how quantitative variables were handled in the analyses. If applicable, describe which groupings were chosen and why | |  |
| Statistical methods | | 12 | | (*a*) Describe all statistical methods, including those used to control for confounding | | Yes |
|  |  |  |  | (*b*) Describe any methods used to examine subgroups and interactions | | Yes |
|  |  |  |  | (*c*) Explain how missing data were addressed | | NA |
|  |  |  |  | (*d*) *Cohort study*—If applicable, explain how loss to follow-up was addressed  *Case-control study*—If applicable, explain how matching of cases and controls was addressed  *Cross-sectional study*—If applicable, describe analytical methods taking account of sampling strategy | | NA |
|  |  |  |  | (*e*) Describe any sensitivity analyses | | Yes |
| Results | | | | |  | |
| Participants | 13* | | (a) Report numbers of individuals at each stage of study—eg numbers potentially eligible, examined for eligibility, confirmed eligible, included in the study, completing follow-up, and analysed | | Yes | |
|  |  |  | (b) Give reasons for non-participation at each stage | | NA | |
|  |  |  | (c) Consider use of a flow diagram | | Yes | |
| Descriptive data | 14* | | (a) Give characteristics of study participants (eg demographic, clinical, social) and information on exposures and potential confounders | | Yes | |
|  |  |  | (b) Indicate number of participants with missing data for each variable of interest | | Yes | |
|  |  |  | (c) *Cohort study*—Summarise follow-up time (eg, average and total amount) | | Yes | |
| Outcome data | 15* | | *Cohort study*—Report numbers of outcome events or summary measures over time | | *YEs* | |
|  |  |  | *Case-control study—*Report numbers in each exposure category, or summary measures of exposure | | *NA* | |
|  |  |  | *Cross-sectional study—*Report numbers of outcome events or summary measures | | *NA* | |
| Main results | 16 | | (*a*) Give unadjusted estimates and, if applicable, confounder-adjusted estimates and their precision (eg, 95% confidence interval). Make clear which confounders were adjusted for and why they were included | | Yes | |
|  |  |  | (*b*) Report category boundaries when continuous variables were categorized | | NA | |
|  |  |  | (*c*) If relevant, consider translating estimates of relative risk into absolute risk for a meaningful time period | | NA | |
| Other analyses | 17 | | Report other analyses done—eg analyses of subgroups and interactions, and sensitivity analyses | | Yes | |
| Discussion | | | | |  | |
| Key results | 18 | | Summarise key results with reference to study objectives | | Yes | |
| Limitations | 19 | | Discuss limitations of the study, taking into account sources of potential bias or imprecision. Discuss both direction and magnitude of any potential bias | | Yes | |
| Interpretation | 20 | | Give a cautious overall interpretation of results considering objectives, limitations, multiplicity of analyses, results from similar studies, and other relevant evidence | | Yes | |
| Generalisability | 21 | | Discuss the generalisability (external validity) of the study results | | Yes | |
| Other information | | | | |  | |
| Funding | 22 | | Give the source of funding and the role of the funders for the present study and, if applicable, for the original study on which the present article is based | | Yes | |

**Section 2: Procalcitonin algorithm**


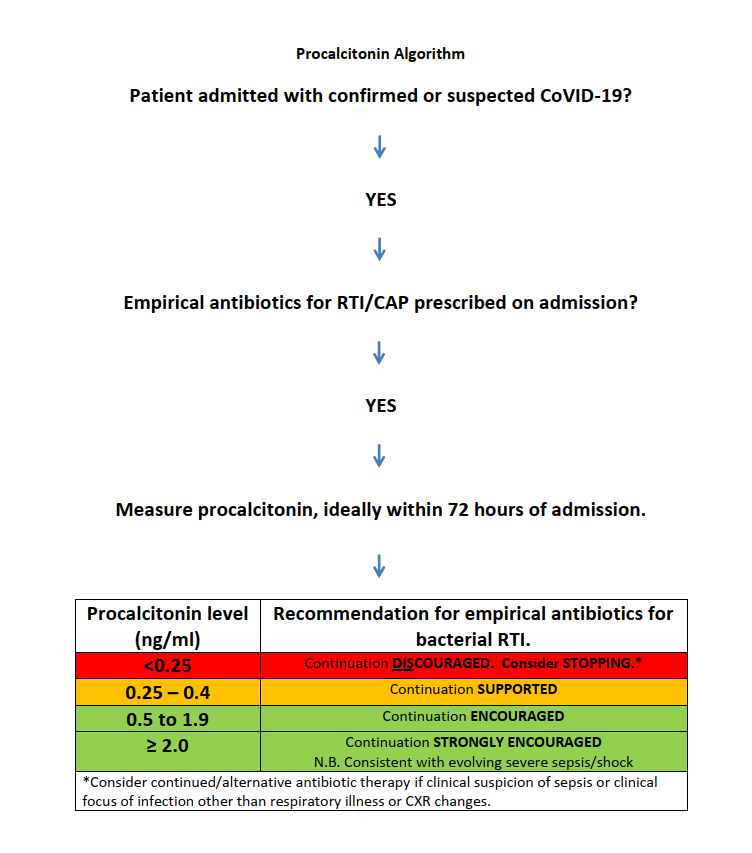


# **Section 3: Supplementary data analysis details**

**Power calculation**

Since our main outcome measure is the difference in antimicrobial exposure, we searched information on this outcome for the sample size calculation. We found a Cochrane systematic review that evaluated this difference in lower respiratory tract infection in 2017.(1) In this paper, the control group had 8.1 ±6.5 days of antimicrobial days exposure and the procalcitonin (PCT) group had 5.6 ±6.5. Using this information, we calculated a minimal sample size needed for comparing two independent means of 116 for each group with power 80% and type I error of 0.05.

**Variables**

Independent variables included were age, sex, comorbidities, observations at admission, presenting symptomatology at admission, chest X-ray changes at admission and COVID-19 status. All were compared between two groups: the procalcitonin group, and the non-procalcitonin group.

Age was analysed as a categorical and continuous variable.

Comorbidities included cardiovascular diseases (hypertension, myocardiopathies, vasculopathies) respiratory conditions (asthma, chronic obstructive pulmonary disease, interstitial diseases), diabetes (type I or type II) and obesity (reported in electronical medical charts or calculated by BMI >30).

Observations at admission included temperature, respiratory rate, oxygen saturation, and oxygen requirement defines as dichotomic variable. We found a collinear relationship between oxygen saturation and oxygen requirements, and thus we only included oxygen requirement in the final model. In contrast there was no collinearity observed for respiratory rate, which was included independently in the modelling.

Presenting symptomatology was defined according to the main complaint of the patient as documented in the medical admission records. Only cough and fever showed differences between groups.

COVID-19 status was confirmed by combined nose and throat swab SARS-CoV-2 PCR assays, taken at any point during hospitalisation.

Dependent variables relating to antimicrobial use were calculated using an Excel spreadsheet (Microsoft Corporation). Timings of antimicrobial doses were defined according to the date and time of drug administration recorded on the patient’s electronic records. The duration of exposure was calculated as the time from first to last dose plus the prescribed dosing interval of the last antimicrobial given. Per-patient consumption was calculated by applying the current World Health Organisation Defined Daily Dose (DDD) method. ^[20]^

Length of stay was recorded in days, rounded up to the nearest integer value.

For safety outcomes, we defined a composite outcome including admission to ICU intra-hospital mortality, mortality at 30 days post admission, and re-hospitalisation within 30 days of index admission. We also individually analysed mortality at 30 days and intra-hospital mortality.

**Analysis Models**

Our primary outcomes (continuous) did not have a normal distribution; this is expected for outcomes of duration and dose / day type, which usually have a positive bias. Homoscedasticity was also not reached. We thus decided to fit and compare both log-gamma and log-normal generalised linear models (GLMs). To determine the optimal model, we compared which GLMs best fits our data using the Akaike information criterion (AIC) and Bayesian information criterion (BIC), and specification tests (Park modified family test, Hosmer-Lemeshow test and Pregibon test), with evaluation of the residuals of each model. Using this approach, the optimal model tfit was achieved using a GLM gamma with link log. In this model, since a logarithmic link is used, each exponentiated coefficient indicates the ratio of the mean of the outcome comparing two individuals that differ in exposure by one unit.

Binary outcomes were compared between procalcitonin and non-procalcitonin groups using prevalence ratios (PRs), which were estimated by fitting robust Poisson models. After discarding collinearity issues, our final model was adjusted for age, sex, comorbidities, COVID-19 status, fever and cough as admission complaint, oxygen requirement and respiratory rate at admission.

**Time Series**

A time series analysis was performed to visualise antimicrobial consumption over time in both groups. Daily antimicrobial consumption (defined daily doses or DDD) by all patients included in the main analysis was averaged at each time point across patients. Time was defined as the date of admission in whole day integer values. Independent time series were then modelled for each group (procalcitonin and non-procalcitonin), fitting a GLM (Gaussian distribution, link identity), treating the DDD-mean as a continuous outcome and time as a predictor. All models were adjusted for autocorrelation and seasonality using Fourier terms (sine and cosine). Presented below is an example of the Stata codes used for fitting the models for procalcitonin (pct==1) and non-procalcitonin (pct==0) groups:

**glm ddd_mean time cos* sin* if pct==0, fam(gaussian) link(identity) glm ddd_mean time cos* sin* if pct==1, fam(gaussian) link(identity)**

Since DDD data was aggregated, no control for confounders was performed. This must be considered when comparing trajectories between procalcitonin and non-procalcitonin groups (i.e. when comparing betas of time between groups). This limitation is discussed in the article.

Reflecting the real-world effect of the availability and use of the procalcitonin assay, a sensitivity analysis was performed by re-including in both groups patients who had discontinued antimicrobials due to palliation at the end of life, using identical models and Stata codes to the main analysis.

# **Table S1: Antimicrobial consumption for treatment of patients with COVID-19 pneumonia in defined daily doses.**

|  | Non-procalcitonin (n=142) | Procalcitonin (n=117) | Total |
| --- | --- | --- | --- |
| Amoxicillin IV | 51.3 | 62.3 | 113.7 |
| Amoxicillin PO | 181.0 | 106.0 | 287.0 |
| Aztreonam IV | 1.3 | 0 | 1.3 |
| Cefuroxime IV | 0.0 | 3.0 | 3.0 |
| Chloramphenicol IV | 6.8 | 8.1 | 14.9 |
| Ciprofloxacin PO | 7.0 | 0.0 | 7.0 |
| Clarithromycin IV | 24.0 | 13.5 | 37.5 |
| Clarithromycin PO | 121.0 | 141.0 | 262.0 |
| Co-amoxiclav IV | 16.7 | 8.3 | 25.0 |
| Co-amoxiclav PO | 63.0 | 22.7 | 85.7 |
| Co-trimoxazole IV | 0.0 | 3.5 | 3.5 |
| Co-trimoxazole PO | 19.5 | 13.3 | 32.8 |
| Doxycycline PO | 413.0 | 209.0 | 622.0 |
| Ertapenem IV | 5.0 | 0.0 | 5.0 |
| Gentamicin IV | 1.5 | 1.2 | 2.6 |
| Levofloxacin IV | 94.4 | 48.3 | 142.7 |
| Levofloxacin PO | 62.5 | 29.0 | 91.5 |
| Meropenem IV | 0.0 | 1.0 | 1.0 |
| Metronidazole IV | 9.0 | 1.0 | 10.0 |
| Metronidazole PO | 0.8 | 0.0 | 0.8 |
| Piperacillin-Tazobactam IV | 118.3 | 122.6 | 240.9 |
| Teicoplanin IV | 0.0 | 2.5 | 2.5 |
| Total | **1196.0** | **796.2** | **1992.2** |

### Table S2: Microbiology results

| **Microbiology sample results** | **Non-procalcitonin (N=142)** | **Procalcitonin (N=117)** |
| --- | --- | --- |
| Patients in which ≥ 1 set of blood cultures sampled | 83 (58.4%) | 92 (78.6%) |
| Patients with positive blood culture | 10 (7.0%) | 10 (8.5%) |
| Organisms in positive blood cultures: |  |  |
| Coagulase negative staphylococci | 7 | 8 |
| *Clostridium* spp. | 1 | 1 |
| *Bacteroides* spp. | 1 | - |
| Unknown (Gram positive rod) | 1 | - |
| *Acinetobacter* spp. | - | 1 |
| Patients in which ≥ 1 bacterial sputum cultures processed | 18 (12.7%) | 23 (19.7%) |
| Patients with positive sputum cultures | 6 (4.2%) | 4 (3.4%) |
| Organisms in positive sputum cultures: |  |  |
| *Streptococcus pneumoniae* | 1 | - |
| *Haemophilus influenzae* | 2 | - |
| *Moraxella catarrhalis* | - | 1 |
| *Klebsiella pneumoniae* | - | 1 |
| *Pseudomonas aeruginosa* | 3 | 2 |
